# Resting-state EEG datasets of adolescents with mild, minimal, and moderate depression

**DOI:** 10.1186/s13104-021-05673-x

**Published:** 2021-07-02

**Authors:** Saravut Rachamanee, Peera Wongupparaj

**Affiliations:** 1 Faculty of Management Sciences and Information Technology, Nakhon Phanom University, Mueang, Thailand; 2 Cognitive Science and Innovation Research Unit (CSIRU), College of Research Methodology and Cognitive Science, Burapha University, Saen Suk, 20131 Thailand

**Keywords:** Eyes-closed and-open resting conditions, Adolescents with depressive symptoms, Absolute and relative EEG powers, Patient Health Questionnaire-9, Beck Depression Inventory-II

## Abstract

**Objectives:**

To measure depressive severity of 85 Thai adolescents by using the the Beck Depression Inventory-II and the Patient Health Questionnaire-9 and to record the resting-state EEG of these participants.

**Data description:**

The current data note provides raw data of behavioral (i.e., group, BDI-II score, and PHQ-9 score) and electrophysiological parameters (i.e., absolute and relative EEG powers over 64 electrode sites) of 30, 27, and 28 participants with minimal, mild, and moderate depression, respectively. These data are especially useful to investigate the behavioral and electrophysiological markers of adolescents with subclinical depression. It can also be utilized in comparative analysis among age groups, and races.

## Objective

The 2019 global burden of disease study investigated 369 diseases and injuries, 286 causes of death, and 87 risk factors across 204 countries revealed depressive disorders were one of the six common causes of health loss in teenage years [1]. Further, subclinical or subthreshold depression in adolescent is rising notably and under-investigated [2]. Several studies had investigated electrophysiological markers of depressive symptoms and suggested that electroencephalography (EEG) seems promising for detecting depression-related symptoms [3]. Resting-state EEG recording is administered during the absence of any kind of stimulus or activities [4]. Specifically, participants are not required to perform any specific task, except eyes-closed and -open instructions. The resting-state EEG has been employed in many studies and this technique is effective in detecting and predicting depressive disorders, more comfortable, and also easy to record in clinical and research settings [5, 6].

Nonetheless, the extant literature on the resting-state EEG and adolescents with subclinical depression (mild, minimal and moderate) is notably scarce [7]. Accordingly, the main objective was to measure depressive symptoms via two standardized tools, that is, the Beck Depression Inventory-II (BDI-II) [8] and the Patient Health Questionnaire-9 (PHQ-9) [9] and to record the resting-state EEG of 85 Thai adolescents. The current dataset is valuable because it contains behavioral and electrophysiological markers of adolescents with subthreshold depression. Researchers may use these parameters to compare among several ages groups, races and genders. A part of the findings based on this EEG dataset was published in Research Methodology & Cognitive Science [10].

## Data description

The data collection was divided into two phases. First, all eligible participants (N = 85) were adolescents (aged between 13 and 22) with depressive symptoms who did not meet the criteria for major depressive disorder after a clinical evaluation by using the Structured Clinical Interview for DSM-5 (SCID-5). The BDI-II and the PHQ-9 were used by experienced psychiatrists to assess levels of depressive symptoms of participants at a local health promoting hospital from 2017–2018. The BDI-II is a self-report inventory that contains 21 items with a range of score from 0 to 63 and widely used to assess the severity of depression symptomatology [8]. The PHQ-9 is a 9-question depression scale with the range of score from 0 to 27 and used to screen for depressive symptoms in patients [9].

Thus, three groups of participants were identified in the current study, that is, 30 participants with minimal (the BDI-II for scores ranging from 0 to 13 and the PHQ-9 for scores ranging from 1 to 6), 27 participants with mild (the BDI-II for scores ranging from 14 to 19 and the PHQ-9 for scores ranging from 7 to 13), and 28 participants with moderate (the BDI-II for scores ranging from 20 to 28 and the PHQ-9 for scores ranging from 14 to 19) depression [8]. The participants were agreed to take part and they completed the consent forms as approved by the College of Research Methodology and Cognitive Science’s Human Research Ethics Committee (RMCS 005/2560).

Second, the resting-state EEG recorded in the current dataset was part of a larger study protocol. The EEG resting state was measured during eyes-closed and -open sessions with each session lasting 30 s. Continuous EEG recordings were made on a Neuroscan Synamps2/RT amplifier (Compumedics Neuroscan, USA) and 10–20 layout 64-channel Quik-cap electrode system. The ground electrode was at AFz with the reference electrodes located at the left and right mastoid (M1 and M2). The electrode impedance was kept below 5 KΩ. All signals were filtered between 0.05 and 100 Hz online bandpass and sampled at 1000 Hz/channel. Further data preprocessing and processing were performed and extracted from the raw EEG signals using Curry 7 software (Neuroscan Inc., USA). The preprocessing pipeline included 1–40 Hz offline filtering, baseline correction, and Independent Component Analysis (ICA) removal of ocular and muscle artifacts. In addition, the EEG recordings were visually inspected to remove other artifacts. The continuous EEG data were segmented into epochs of 2 s non-overlapping Hanning-windowed epochs. The segmented and accepted EEG epochs absolute (uV2) data were smoothed using Fast Fourier Transform (FFT) and averaged over five frequency bands: delta (1–4 Hz), theta (4–8 Hz), lower alpha (8–10 Hz), upper alpha (10–12 Hz), and beta (13–30 Hz). The absolute and relative EEG powers for five frequency bands were calculated. Accordingly, the current dataset contains two main information, that is, participant characteristics (i.e., group, gender, BDI-II score, and PHQ-9 score) and the absolute and relative EEG powers during eyes-closed and -open sessions over 64 electrode sites from 85 adolescents as can be seen in Data file 1–3 (Table 1).

**Table 1:** Overview of the data files/data sets

Label	Name of data file/data set	File types (file extension)	Data repository and identifier (DOI or accession number)
Data file 1	Participant characteristics	MS Excel file (.xlsx)	Open Science Framework: https://doi.org/10.17605/OSF.IO/4HQ3Y [11]
Data set 1	Eyes-closed EEG	MS Excel file (.xlsx)	Open Science Framework: https://doi.org/10.17605/OSF.IO/4HQ3Y [11]
Data set 2	Eyes-open EEG	MS Excel file (.xlsx)	Open Science Framework: https://doi.org/10.17605/OSF.IO/4HQ3Y (11)

## Limitations

The current dataset aggregated the behavioral and electrophysiological data from adolescents with subclinical depression which is limited in generalizability. Additionally, the EEG dataset includes a large sample, but there were substantially more women than men (65% vs 35%). Thus, caution is needed in analyzing and interpreting results.

## Data Availability

The data described in this data note can be freely and openly accessed via the Open Science Framework (OSF) (10.17605/OSF.IO/4HQ3Y). Please see Table 1 for details of the links to the data.

## Abbreviations

EEG: Electroencephalogram
BDI-II: Beck Depression Inventory-2nd edition
PHQ-9: Patient Health Questionnaire-9
ICA: Independent Component Analysis
FFT: Fast Fourier Transform
